# Whole genome annotation and comparative genomic analyses of bio-control fungus *Purpureocillium lilacinum*

**DOI:** 10.1186/s12864-015-2229-2

**Published:** 2015-11-25

**Authors:** Pushplata Prasad, Deepti Varshney, Alok Adholeya

**Affiliations:** TERI Deakin Nanobiotechnology Centre, TERI Gram, The Energy and Resources Institute, Gual Pahari,Faridabad Road, Gurgaon, Haryana 122 001 India

**Keywords:** *P. lilacinum*, *de novo* assembly, Whole genome sequencing, Annotation, Pathogen-host interaction, Phylogenomic analysis

## Abstract

**Background:**

The fungus *Purpureocillium lilacinum* is widely known as a biological control agent against plant parasitic nematodes. This research article consists of genomic annotation of the first draft of whole genome sequence of *P. lilacinum*. The study aims to decipher the putative genetic components of the fungus involved in nematode pathogenesis by performing comparative genomic analysis with nine closely related fungal species in Hypocreales.

**Results:**

*de novo* genomic assembly was done and a total of 301 scaffolds were constructed for *P. lilacinum* genomic DNA. By employing structural genome prediction models, 13, 266 genes coding for proteins were predicted in the genome. Approximately 73 % of the predicted genes were functionally annotated using Blastp, InterProScan and Gene Ontology. A 14.7 % fraction of the predicted genes shared significant homology with genes in the Pathogen Host Interactions (PHI) database. The phylogenomic analysis carried out using maximum likelihood RAxML algorithm provided insight into the evolutionary relationship of *P. lilacinum*. In congruence with other closely related species in the Hypocreales namely, *Metarhizium spp*., *Pochonia chlamydosporia*, *Cordyceps militaris*, *Trichoderma reesei* and *Fusarium spp*., *P. lilacinum* has large gene sets coding for G-protein coupled receptors (GPCRs), proteases, glycoside hydrolases and carbohydrate esterases that are required for degradation of nematode-egg shell components. Screening of the genome by Antibiotics & Secondary Metabolite Analysis Shell (AntiSMASH) pipeline indicated that the genome potentially codes for a variety of secondary metabolites, possibly required for adaptation to heterogeneous lifestyles reported for *P. lilacinum*. Significant up-regulation of subtilisin-like serine protease genes in presence of nematode eggs in quantitative real-time analyses suggested potential role of serine proteases in nematode pathogenesis.

**Conclusions:**

The data offer a better understanding of *Purpureocillium lilacinum* genome and will enhance our understanding on the molecular mechanism involved in nematophagy.

**Electronic supplementary material:**

The online version of this article (doi:10.1186/s12864-015-2229-2) contains supplementary material, which is available to authorized users.

## Background

*Purpureocillium lilacinum*, previously named as *Paecilomyces lilacinus*, belongs to the fungal order Hypocreales that harbors species known to produce a diversity of secondary metabolites and bio-actives [1]. *P. lilacinum* produces paecilotoxin and is considered as a significant biological control agent against plant parasitic nematodes, especially economically important species of *Meloidogyne incognita* [2, 3]. Diseases caused by plant-parasitic nematodes are considered as one of the major threats for global food security [4–7]. Nematode infection in economically important crops results in an estimated annual loss of US $100 billion [8]. Research on nematode-suppressive soils has revealed a substantial presence of nematophagous fungi *P. lilacinum* and *Pochonia chlamydosporia* [9, 10]. Similar to other species in Hypocreales, *P. lilacinum* has a broad host range and its various strains parasitize different species of nematodes and insects [11]. In addition, endophytically-colonized *P. lilacinum* has been recently reported to adversely affect reproduction of cotton-aphids putatively by inducing systemic response in plant [12].

The fungal order Hypocreales consists of plant-pathogens, insect-pathogens, nematode-pathogens, plant-endophytes and mycoparasites. These fungi are multi-trophic in nature and several transitions between lifestyles have been predicted in their evolutionary history [13–15]. *P. lilacinum* is a ubiquitous soil hyphomycete and carry out saprophytic activities in varied habitats including agricultural fields, forests, grassland, deserts and estuarine sediments. Different isolates of *P. lilacinum* are found resistant to a wide range of temperature and pH [16]. On the other hand, *P. lilacinum* is also reported to exercise parasitic or endophytic lifestyles in the presence of a host organism such as nematodes, aphids and cotton plants (Gossypium hirsutum) [9–12].

*P. lilacinum* infects eggs and females of *Meloidogyne spp*. and causes death of the nematode embryos in 5 to 7 days [17]. A strain of *P. lilacinum*, strain 251, is now an active ingredient in several commercial bio-nematicides [18]. However, the molecular basis of the pathogenic mechanism employed by *P. lilacinum* against nematodes has been meagerly elucidated till date. Whole genome sequencing efforts of other Hypocreales fungi [1, 13, 19–22] have revealed that these genomes code for an array of hydrolytic enzymes, secondary metabolites/bioactives, non-ribosomal protein synthetases (NRPS) and polyketide synthetases (PKS) that are essential for their bio-control attribute. Experimental evidences show that extracellular hydrolytic enzymes including proteases, collagenases and chitinases are involved in the degradation of egg-shell components by *P. lilacinum* [23, 24]. Despite of strong prospect of secondary metabolites and hydrolytic enzymes produced by *P. lilacinum* in biological control of phyto-pathogens, only one gene encoding for serine protease [23] and one encoding for keratinase [16] have been molecularly characterized so far.

Considering the increasing demand for alternative plant-pathogen management approaches in agricultural systems, we carried out whole genome annotation and comparative genomic analyses of the nematophagous fungus *P. lilacinum*. The study provides new insights into the genome of this commercially important biological control agent. The work would pave way for understanding important pathways and genes utilized by *P. lilacinum* to carry out nematophagous and endophytic activities, which is expected to aid in developing methods to protect crop plants.

## Results and discussion

### Global genome structure

The *P. lilacinum* genome was sequenced to 200 x coverage and by mapping the reads to contigs, the genome sequence was assembled into 301 scaffolds with total gap size of 526,913 bases. The sum of the scaffolds length is equal to 40.02 Mb. The longest scaffold length was of 3.7 Mb. The N50 and N90 values were 1,827,308 bp and 93,833 bp respectively. The predicted assembly size was 44.76 Mb by k-mer analysis with the best kmer value = 51 (Additional file 1: Figure S1). The MAKER annotation pipeline [25] predicted 13,266 protein coding genes, which is comparatively more than other species belonging to entomopathogenic or mycoparasitic families of Hypocreales. Core Eukaryotic Genes Mapping (CEGMA) [26, 27] analysis against a set of 248 conserved protein families that occur in a wide range of core eukaryotic gene datasets (CEGs) http://korflab.ucdavis.edu/Datasets /genome completeness/index.html#SCT2) found 96.77 % of the core genes were matched, indicating that the draft genome sequence of *P. lilacinum* was largely complete (Additional file 1: Table S1). tRNAScan-SE [28, 29] predicted a total of 91 tRNAs in the genome. The average gene density (303/Mb) and the average gene length (1.51 Kb) of *P. lilacinum* are similar to other Ascomycetous fungi [13]. The genome size (40.02 Mb), total number of predicted genes (13, 266) and the %GC (58.57 %) content of *P. lilacinum* are comparable to the nematophagous fungus *Pochonia chlamydosporia* (41.0 Mb) of Hypocreales (Table 1 and Additional file 1: Table S2). When compared with other fungal orders, the genome size of *P. lilacinum* is also similar to that of *Trichaptum abientinum* (40.61 Mb) belonging to the order Polyporales in phylum Basidiomycota and *Melanomma pulvi-pyris* (42.09 Mb) belonging to the order Pleosporales in Phylum Ascomycota [30].

**Table 1:** Genomic features of *P. lilacinum*

Features	*P. lilacinum*
Genome Size (Mb)	40.02
%GC content	58.57 %
Predicted Proteins	13266
Avg. Gene Density(genes/Mb)	303
Avg. Gene length (bp)	1512
Repeat Content %	1.68 %
tRNAs	91
Secreted Proteins	1276
Secondary metabolites clusters	30 (SMURF), 46 (AntiSMASH)
PHI genes	1953
Proteases	480

### Mobile elements

A 1.68 % fraction of the *P. lilacinum* genome was estimated to consist of repeated sequences. A total of 105 retrotransposons (class I), 2 DNA transposons (class II), and 1 unknown element (Additional file 1: Table S3) were identified in the genome. The number and family wise distribution of mobile elements in *P. lilacinum* genome is comparable other insect pathogenic fungal species in Hypocreales [19–22].

### Gene Ontology (GO) based distribution of genes

**Fig. 1:**
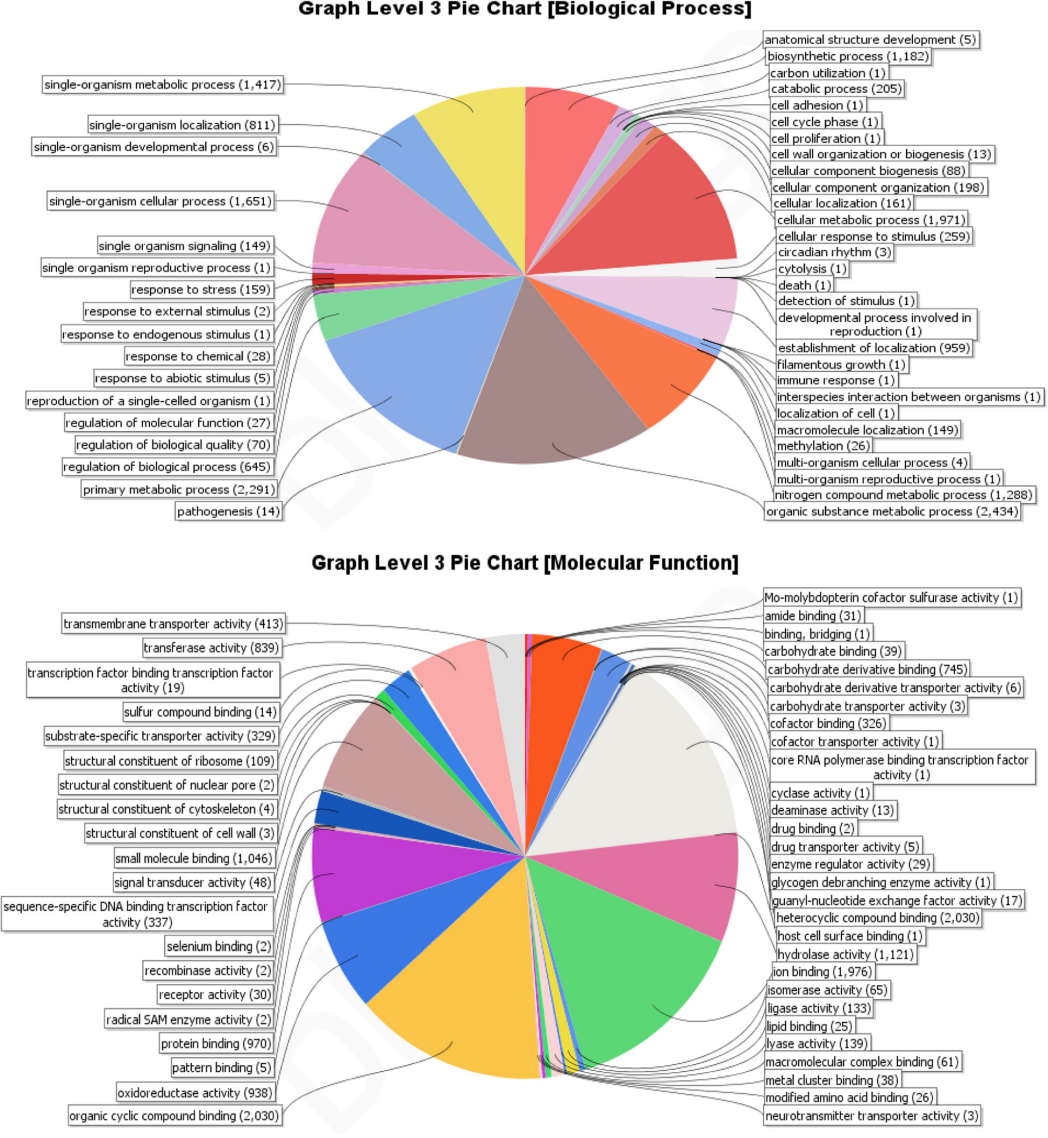
GO based functional annotation of genes present in the *P. lilacinum* genome. Biological Process domains and Molecular function domains

Of the total predicted genes in *P. lilacinum*, 9800 (73 %) had Blastp hits and 8204 proteins (62 %) had InterProScan hits. Genes having InterProScan hits were investigated for distribution into functional categories based on Gene Ontology (GO). Allocation by GO domains, “Biological Process” and Molecular Function”, according to generic terms at level 3 in Blast2GO is presented in Fig. 1. Major “Biological process” groups were constituted by the following GO terms: organic substance metabolic process (18 %), primary metabolic process (17 %), biosynthesis process (9 %), establishment of localization (7 %), catabolic process (1.5 %), response to stress (1 %), and pathogenesis genes (0.1 %). Distribution according to “Molecular function” largely encompassed those genes implicated in heterocyclic compound binding (15 %), ion binding (14 %), hydrolase and lyase activity (9.5 %), oxidoreductase activity (7 %), small molecule binding (7.8 %), protein binding (7.3 %), transferase activity (6 %), carbohydrate and carbohydrate derivative binding (5.6 %) and trans-membrane transporter activity (3.1 %).

### Phylogenomic relationships

*Neurospora crassa* belongs to the order Sordariales. The orders Hypocreales and Sordariales belong to the fungal class Sordariomycetes under Ascomycetes. In our phylogenomic analysis *Neurospora crassa* served as an out group. Orthologous protein clusters generated by MCL clustering tool embedded in the phylogenomic pipeline Hal [31] were sieved to get non-redundant clusters. Aligned clusters were concatenated to generate super-alignments that were used to build maximum likelihood phylogeny tree.

**Fig. 2:**
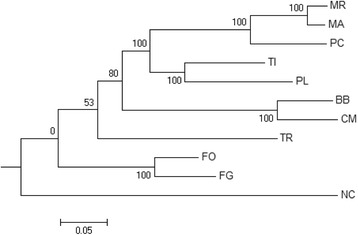
Phylogenetic tree of *P. lilacinum*

The constructed phylogenomic tree (Fig. 2) mimicked the recent taxonomic classification with the representative species belonging to the five families in Hypocreales being clustered under a separate node from *Neurospora crassa.* The family Nectriaceae comprising primarily of plant-pathogens such as *F. graminearum* and *F. oxysporum* diverged the earliest. Members of Hypocreaceae (represented by *Trichoderma reesei* in this analysis) are widely known to possess mycoparasitic life style. Hypocreaceae composed a sister node to Cordycipitaceae family that holds insect-pathogenic fungal species *Beauveria bassiana* and *Cordyceps militaris*. The family Clavicipitaceae, which by and large includes pathogens of insects, was represented by *Metarhizium robertsii, Metarhizium acridum* and a nematophagous fungus *Pochonia chlamydosporia* in this study*.* Members of Ophiocordycipitaceae included *Tolypocladium inflatum* and *P. lilacinum*. *T. inflatum* is a pathogen of beetle larvae and *P. lilacinum* is a nematophagous fungus. Cordycipitaceae and Ophiocordycipitaceae seemed to have diverged earlier in comparison to Clavicipitaceae in the evolutionary history. Genome sequencing data of the species belonging to the remaining two families Niessliaceae and Bionectriaceae in Hypocreales has not been released as yet in the public domain by DOE (Department of Energy, http://genome.jgi-psf.org/) and therefore, not included in the analysis.

Phylogenomic analysis presented in this study was largely in agreement with typical phylogenetic studies that have sampled a large number of Hypocreales species and constructed evolutionary relationships from meta-analysis of multi-gene datasets [14, 15, 32]. However, taxonomic refinement of Clavicipitaceae, which was grouped into Clavicipitaceae A, B and C by multi-gene phylogenetic analysis, could only be achieved by phylogenomic analysis done using genome-scale data that inferred polyphyletic origin of taxons with entomopathogenic fungi in three separate families in Hypocreales [33, 34]. Our results were in absolute confirmation with a previous report that presented an elaborate account of phylogenomic relationships of Hypocreales [13]. Our phylogenomic analysis indicated lifestyle transitions among the different species in the Hypocreales from plant-pathogens in initially diverging taxon (Nectriaceae) to mycoparasitism (Hypocreaceae) and entomopathogenic trophic behaviors (Cordycipitaceae, Ophiocordycipitaceae and Clavicipitaceae).

Taxonomic placement of *P. lilacinum* remained ambiguous until recent. The fungus was previously placed under Trichocomaceae that contains the very familiar genus *Penicillium*. Genome-scale phylogeny constructed in this study clearly places *P. lilacinum* among taxons with insect-pathogens and under the family Ophiocordycipitaceae along with *T. inflatum.*

### Potential pathogenesis-related genes

To find potential pathogenesis-related genes, a whole genome blast analysis was conducted against the pathogen-host interaction (PHI) gene database version 3.6 [35] at E < 1*10^−20^. PHI db catalogs experimentally validated pathogenic, virulence and effector genes from fungal, Oomycete and bacterial pathogens of fungi, insects, plants and animals hosts. Homolog identification in PHI db, therefore, indicates towards putative role of the gene in pathogenesis. Approximately 14.7 % (*N* = 1953 genes) of the protein-coding genes with Blastp hits in the genome of *P. lilacinum* showed homology with the genes present in the PHI db.

Total number of genes sharing homology with PHI db in the 10 Hypocreales genomes analyzed in this study is presented in Additional file 1: Table S4. Using Blastp (E < 1*10^−20^) and PHI version 3.6, highest number of PHI homologous genes were observed in plant pathogens *F. oxysporum* (2486 genes), which was followed by *P. lilacinum* (1953 genes). The insect pathogens *M. acridum* and *M. robertsii* were found to possess 1477 and 1593 genes respectively. Surprisingly, the genome of the other nematophagous fungus *P. chlamydosporia* contained only 1402 PHI homologs as compared to 1953 homologs in *P. lilacinum*.

Genes in PHI db (ver 3.6) were further sieved to select a set of genes that were experimentally validated for their role in pathogenicity by knock-out experiments. Blastp search revealed presence of 469 (3.53 %) homologs to “loss of pathogenicity genes”, 92 (0.69 %) “increased virulence genes”, 1311 (9.9 %) “reduced virulence genes”, 40 (0.3 %) “effector plant virulence genes”, 303 (2.28 %) “mixed pathogenesis genes” and 226 (1.70 %) “lethal genes” in the *P. lilacinum* genome.

**Fig. 3:**
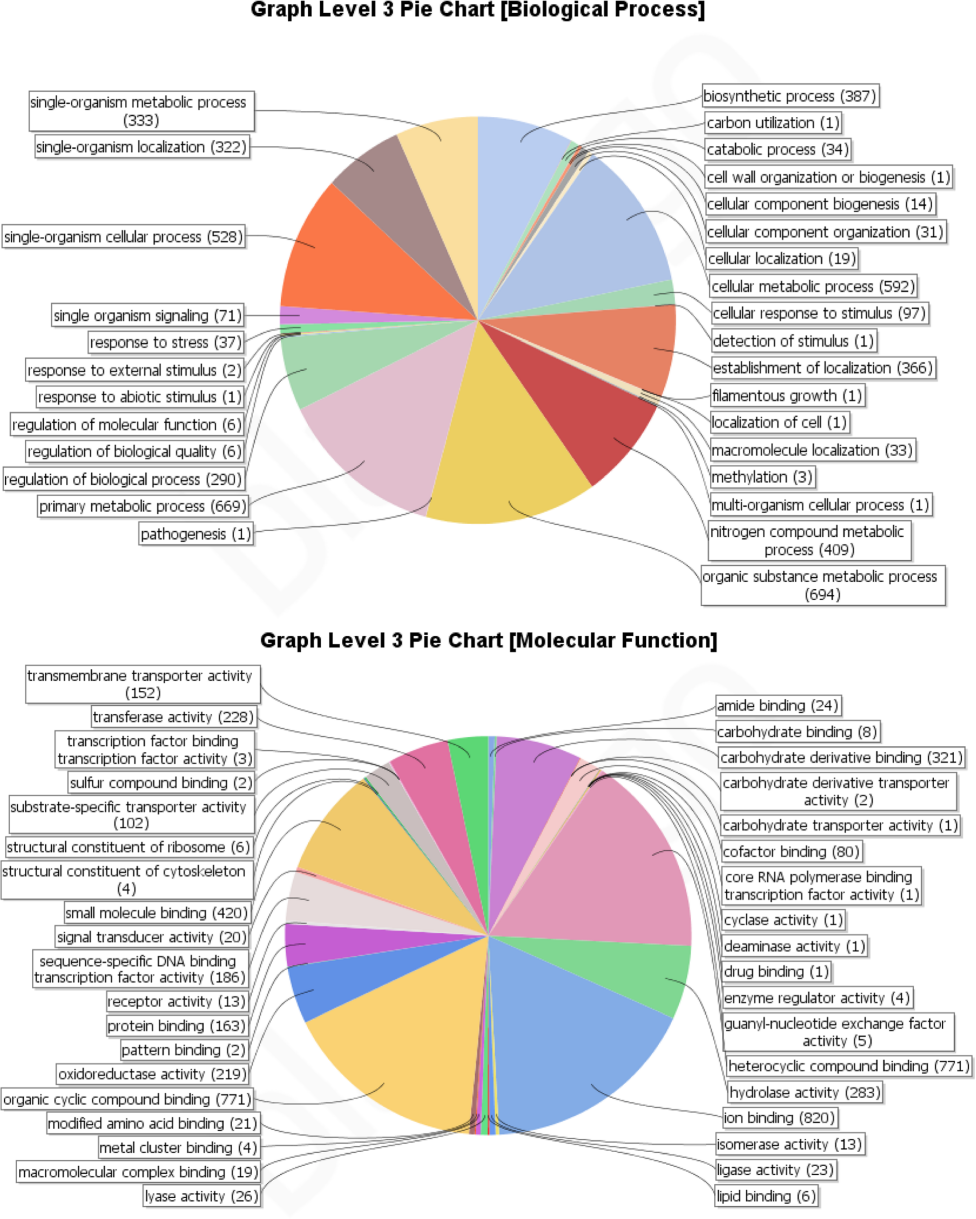
GO based functional annotation of genes with homologs in PHI db. Biological Process domains and Molecular function domains

To identify the putative involvement of the pathogenesis related genes in biological and molecular processes, Blast2GO based annotation was carried out. Based on presence of the GO domains, PHI genes were grouped into 30 biological-functional groups and 37 molecular-function groups (Fig. 3). Major biological process groups included genes related to cellular metabolic processes (30 %), primary metabolism (34 %), biosynthetic processes (19.81 %), regulation of biological processes (14 %), establishment of localization (18 %) and response to stress (1.5 %). However, unlike 40 PHI genes in *P. chlamydosporia*, only one PHI gene was found under GO term “pathogenesis” in *P. lilacinum*. Fisher exact test was done for statistical evaluation of difference in genetic make-up between *P. lilacinum* and *P. chlamydosporia* with respect to important genes. Additional file 1: Table S5 shows that significantly different proportion of establishment of localization, primary metabolic process, glycoside hydrolases, secondary metabolites, response to stress genes were present in *P. lilacinum* as compared to *P. chlamydosporia*, with abundance of serine proteases in *P. lilacinum.*

These findings probably indicate towards differences in the genetic arsenals between the two nematophagous fungi, *P. lilacinum* and *P. chlamydosporia,* belonging to two different families Ophiocordycipitaceae and Clavicipitaceae respectively of Hypocreales. Fewer matches with GO term “relationship between organisms” was not unexpected as only 14 genes in the complete genome could be identified under the category “pathogenesis” in Blast2GO based annotation of the genome (Fig. 1). Interestingly, of the 1, 259 protein-coding genes identified as associated with the GO terms “hydrolase activity” and “lyase activity” under “Molecular Function” domain in whole genome analysis (Fig. 1), 283 hydrolase and 26 lyase activity signatures were found in the PHI db, which is suggestive of their importance in pathogenesis (Fig. 3).

### Secreted proteins in *P. lilacinum*

The secreted proteome plays a crucial role in defining the ability of a fungal pathogen to interact with the host and the environment. Using the online tool SignalP (ver 4.1), a total of 1,276 secreted peptides were predicted in *P. lilacinum* genome, of which 184 had homologs in PHI db (Additional file 1: Table S6). The number of secreted proteins reported for the insect pathogens *M. robertsii* and *M. acridum* [20, 21] and plant pathogens [36] and non-pathogens [37] are estimated using SignalP (ver 3) instead of the recent version SignalP (ver 4.1) and therefore, a comparison between *P. lilacinum* genome and the remaining species is difficult. Considerably large assimilation of secretary proteins in *P. lilacinum* genome could indicate towards increased complexity of secreted proteome for improved adaptation to environment.

### Signal transduction and gene regulation

Multiple lifestyles acquired by the fungus *P. lilacinum* demands for swift adjustments to varied environments and host. Genes involved in signal transduction and gene regulation are crucial in lifestyle transitions. Fungal G-protein coupled receptors (GPCRs) are required for transducing environmental cues, which involve niche recognition, nutrient sensing and recognition of host immune system [34, 38]. In pathogenic fungi *Parastagonospora nodorum*, alpha subunits of G protein are found important in causing plant-disease by transducing extracellular signals [39]. In *Magnaporthe sp.,* the Pth11-like GPCR triggers cell differentiation in response to plant inductive cues and mediates pathogenicity [40]. *P. lilacinum* genome encoded 33 GPCRs, compared to an average of 32 in other fungi [20]. 12 GPCRs shared homology with Pth11-like GPCRs (Additional file 1: Table S7). Number of total GPCRs and Pth11-like GPCRs in *P. lilacinum* genome are comparable to that of *C. militaris* [1] but much lesser than sequenced Clavicipitaceae fungi, *Metarhizium spp*. [20].

Histidine kinases (HK) are largely implicated in environment sensing and stress responses in fungi [41]. *P. lilacinum* possesses 24 HK proteins compared to 17 in *M. robertsii* and 9 in *M. acridum*. Substantially increased number of HKs could indicate towards efficient signal transduction capacity of the multi-trophic fungi, *P. lilacinum*, under rapidly changing environmental conditions.

**Fig. 4:**
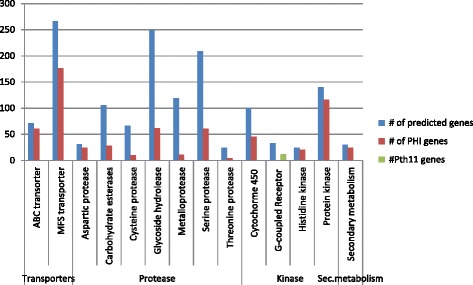
Gene families important in nematode pathogenesis and their homologs in PHI db

Protein kinases (PKs) are important in regulation of cellular and metabolic processes primarily signal transduction. 140 PK genes were identified in *P. lilacinum* as compared to 153 in *Pochonia chlamydosporia*, 167 in *C. militaris*, 158 in *M. robertsii* and 192 in *M. acridum*. Presence of homologs for a significant subset (116 genes) of PKs in PHI db emphasizes possibility of these genes in pathogenicity. Number of signal transduction genes and their homologs in PHI db has been shown in Fig. 4.

### Genes involved in transportation and detoxification of compounds

In pathogenic and parasitic fungi, ATP-binding cassette (ABC) transporters are largely implicated in defense mechanism and protect the fungus from secondary metabolites or toxins produced by the host [42]. The *P. lilacinum* genome coded for 71 ABC transporters, of which 61 had homologous gene present in the PHI db. Another important family of transporters known as major facilitator super-family (MFS) is frequently involved in the transport of a wide range of substrates and nutrient sensing [42]. The genome of *P. lilacinum* encoded 266 MFS transporters, of which 176 genes had homologs in PHI db.

Cytochrome P450 (CYP) monooxygenase superfamily mediates numerous functions in fungi. Most importantly, CYPs contribute in multifaceted metabolism and adaptation to varied ecological niches [43]. Filamentous fungi produce an array of secondary metabolites, biosynthesis of many being mediated by CYPs [44, 45]. CYPs are also known to contribute towards pathogenicity and life style of fungi. In *P. lilacinum* 100 CYP genes were identified, of which only 45 exhibited homologous counterparts in the PHI db. Number of CYP enzymes in *P. lilacinum* is similar to Clavicipitaceae fungi (*P. chlamydosporia*, *M. robertsii* and *M. acridum*) but is more than previously reported plant pathogenic (*F. graminearum*, *M. oryzae*) fungi. Additional file 1: Table S8 enlists Pth11 like GPCRs, Histidine kinases, Protein kinases, ABC, MFS and CYP genes in *P. lilacinum*. Number of transportation & detoxification genes and their homologs in PHI base has been shown in Fig. 4.

### Hydrolytic enzymes

To be a successful parasite, saprophyte and endophyte, *P. lilacinum* codes for an array of hydrolytic enzymes including proteases, chitinases, lipases and esterases. The size of hydrolytic enzyme families with putative biological function and number of homologs in PHI db has been presented in Fig. 4. Our analysis of the *P. lilacinum* genome unraveled the likely genetic requirements to invade the egg shells of nematodes by disintegration of egg shell components.

### Proteases

By carrying out a Blastp (batch Blast) search against the MEROPS protease database [46, 47], we identified 480 genes coding for proteases in the *P. lilacinum* genome. According to their catalytic type these proteases were divided into five categories, which were further distributed into 72 families (Additional file 1: Table S9). Similar to other entomopathogenic fungi, serine proteases with 209 genes constituted the largest category of proteases in *P. lilacinum,* the three major classes being prolyl oligopeptidases (S09; 69 proteins), prolyl aminopeptidases (S33; 53 proteins) and subtilisins (S08; 40 proteins, S53; 9 proteins). Subtilisin-like serine proteases produced by *P. lilacinum* have been identified to degrade protein components of both nematode and insect eggs [48]. In addition, reports indicate that subtilisin-like serine proteases could have been crucial during the evolution of pathogenicity of nematode-trapping fungi against nematodes [48]. In nematophagous fungus *Pochonia chlamydosporia*, in addition to subtilisins, serine carboxypeptidases are also instrumental in the penetration of nematode egg shell [49]. 59 % of serine proteases showed expression under endophytism in *P. chlamydosporia* [22]. The *P. lilacinum* genome coded for 35 serine proteases of S8 family (subtilisins), 9 serine proteases of S53 (subtilisins) and 11 serine proteases of S10 family (serine carboxypeptidases). Expression of 50 % of genes belonging to S10 family during root endophytism in *Pochonia chlamydosporia*, underscored the importance of serine carboxypeptidases in disintegration of the nematode egg shell. In our study, we found that 61 genes coding for serine proteases had homologous present in the PHI db (Additional file 1: Table S10), of which 15 were subtilisins (S08) and 9 were serine carboxypeptidases(S10), which further supported their role in pathogenicity. Similar to *P. chlamydosporia*, the second largest group of proteases in *P. lilacinum* genome was made of Metalloproteases, with 119 genes spread over 25 families. The largest family within Metalloproteases was glutamate carboxypeptidases (M20; 18 genes). The important classes of metalloproteases that were reportedly expressed during endophytism in *P. chlamydosporia* were fungalysins (M36), carboxypeptidases (M28) and deuterolysins (M35). The *P. lilacinum* genome harbored 2 fungalysins, 11 carboxypeptidases and 7 deuterolysins quite in an adequate proportion with *Pochonia chlamydosporia* genome. Remaining proteases in the genome belonged to threonine protease class with 24 genes, cysteine proteases with 66 genes and aspartic proteases with 31 genes.

### Glycosides hydrolases and carbohydrate esterases

The egg-shell components of nematodes include structural polysaccharides such as cellulose, chitin and mannan. In order to penetrate the nematode egg, *P. lilacinum* needs to produce glycoside hydrolases (GH) and carbohydrate esterases (CE) for degradation of polysaccharides. Using CAZy database [50] and performing HMMER [51] scan based on the profile compiled in dbCAN release 2.0; we predicted presence of 249 glycoside hydrolases (GH) and 106 carbohydrate esterases (CE) in *P. lilacinum* genome. The size of the GH family in *P. lilacinum* is very close to *F. graminearum* (269 genes) of Nectriaceae and *P. chlamydosporia* (281 genes) of Clavicipitaceae, as compared to small sized family in *M. acridum* (176 genes) and *M. robertsii* (194 genes) of Clavicipitaceae (Additional file 1: Table S11). Chitinases coded by 28 genes of GH18 class were the most abundant of all glycoside hydrolases in *P. lilacinum*. 13 genes encoding for cellulases belonging to GH5–GH12 families were identified in this genome. Cellulases were found to be expressed during root colonization in *P. chlamydosporia* and thus could be important in life style transitions in *P. lilacinum*.

Carbohydrate esterases (CEs) carry out the de-O or de-N-acylation of esters or amides and other substituted saccharides, in which sugars play the role of alcohol and amine. Our analysis showed that Hypocreales fungi (*P. lilacinum*, *P. chlamydosporia, F. graminearum* and *Metarhizium spp.*) have genes from 15 of the 16 CE families, with CE15 being the only missing family (Additional file 1: Table S12). We identified 106 genes encoding carbohydrate esterase enzymes in *P. lilacinum*, with CE10 being the largest family. Members of families CE1 and CE10 possessed maximum number of genes coding for carbohydrate esterases. CE1 and CE10 share the common activities of carboxylesterase and endo-1,4-β-xylanase [52]. However, they greatly differ in substrate specificity. The number of CE enzymes in *P. lilacinum* genome was more than that reported for Clavicipitaceae family and less than Nectriaceae family under the Hypocreales order.

In *P. lilacinum* 62 GH and 28 CE genes had homologs counterparts in PHI db (Additional file 1: Table S13). The genome seems to be enriched with hydrolytic enzymes and employs an assortment of proteases, glycoside hydrolases, and carbohydrate esterases to breach the egg-shell barrier in order to parasitize nematode.

### Secondary metabolite genes and clusters

Species in Hypocreales are known for production of secondary metabolites that include toxins and pharmaceutically active components. *P. lilacinum* is widely known to produce a leucinostatin named as paecilotoxin. Leucinostatins are linear nine residue peptabiotics, characterized by N-terminal proline acetylated by MeHA and C-terminal B-Ala amidated by DPD. Leucinostatin is ubiquitously present among all the *P. lilacinum* isolates and its production does not correlate with the habitat or host of the different strains [53]. Production of secondary metabolite other than paecilotoxin has not been reported to date from *P. lilacinum*. Genetic analysis to unveil gene clusters for production of different types of secondary metabolites would help in identification of important bio-actives.

The genes responsible for biosynthesis, export, and transcriptional regulation of secondary metabolites are often found in contiguous gene cluster. Secondary metabolite unique regions finder (SMURF) is a web-based tool [54] that finds secondary metabolite biosynthesis gene clusters and pathways in fungal genomes based on their genomic context and domain content. Another *in silico* pipeline antiSMASH 2.0 [55], identifies bio-synthetic loci coding for secondary metabolites including oligosaccharide antibiotics, phenazines, homoserine lactones, thiopeptides, furans and phosphonates, in addition to secondary metabolite classes: polyketides, non-ribosomal peptides, terpenes, aminoglycosides, aminocoumarins, indolocarbozoles, lantibiotics, bacteriocins, nucleosides, beta-lactams, butyrolactones, siderophores, melanins. Using SMURF, 30 secondary metabolite genes were identified (Additional file 1: Table S14). These genes included 12 PKS (polyketide synthase), 2 PKS-like enzymes, 7 NRPS (nonribosomal polyketide synthase), 7 NRPS-like enzymes, 1 hybrid and 1 DMAT (dimethyllacetyltransferase). In comparison to other sequenced fungi in Hypocreales, SMURF analysis indicated that *P. lilacinum* has lesser capacity for the production of bio-actives and toxins. However, antiSMASH run on the genome data identified 46 clusters (10 nrps, 16 type 1 pks, 9 terpene, 1 lantipeptide,1 linaridin, and 9 other secondary metabolites clusters), which is close to the maximum (51) reported in entomopathogenic fungus *M. robertsii* [20, 21]. AntiSMASH based Functional annotations of the 46 clusters are presented in (Additional file 1: Table S15). AntiSMASH analysis suggested that *P. lilacinum* could encode several non-conventional secondary metabolites by using alternate gene clusters.

Bioinformatics tool Natural Product Domain Seeker (NaPDoS) (http://napdos.ucsd.edu) (Db version: pksdb_20111206) was used to detect and extract condensation (C) and ketosythase (KS) domains through a broad set of manually curated reference genes from well-characterized chemical pathways. We found 18 PKS-derived KS domains and 10 NRPS-derived C- domain in *P. lilacinum* (Additional file 1: Table S16).

### Quantitative real-time PCR

**Fig. 5:**
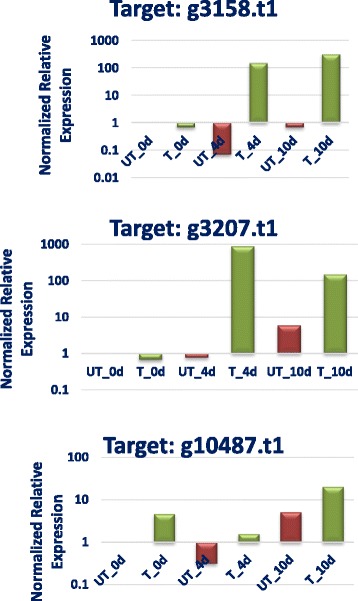
Relative quantitative gene expression for subtilisin-like serine proteases in *P. lilacinum* in presence and absence of the nematode host (*M. incognita*) eggs

To validate importance of serine proteases expressed by *P. lilacinum* in nematode pathogenesis, quantitative real-time PCR analyses were carried out. Three subtilisin-like serine proteases, namely, g3158.t1, g3207.t1, and g10487.t1, were selected as target genes for the analyses and actin gene was chosen as reference gene. Real-time PCR results confirmed up-regulation of all three subtilisin-like serine proteases and showed that, in the presence of eggs of nematode host (M*eloidogyne incognita*), the copy number of the target genes of *P. lilacinum* was increased in comparison to control (fungus grown in absence of the host) Fig. 5. Notably, relative expression of g3158.t1, g3207.t1 genes was significantly higher (with expression increasing from 148.0561 to 885.2861-folds) on 4th and 10th day post inoculation of *P. lilacinum* with the host nematode eggs. The outcome of quantitative real-time PCR analyses underscore potential role of these subtilisin-like serine proteases in nematode egg shell degradation.

## Conclusions

The whole genome annotation and comparative genomic analyses of the first draft genome sequence of monophyletic genus *Purpureocillium lilacinum* provided insight into its genetic make-up and phylogenetic placement. Presence of a variety of hydrolytic proteins in the genome illustrated putative genetic machinery involved in nematophagous activity. Quantitative real-time PCR analyses corroborated role of serine proteases in nematode pathogenesis. Identification of a plethora of GPCRs, transporter proteins, CYPs and protein and histidine kinase proteins indicate the tentative genetic components important for life style transitions and substantiate the observed multi-trophic behaviour viz., saprophyte, egg-parasite, and plant-endophyte of *P. lilacinum*. Sizable representation of important enzyme families in pathogen-host interaction database corroborates importance of the identified proteins in nematode pathogenesis. Significantly large number of secondary metabolite clusters identified through antiSMASH underscores immense capacity of *P. lilacinum genome* to synthesize a variety of typical and atypical secondary metabolites. The gene lists for various protein families provided here, would be useful for future experiments. In addition, phylogenomic study involving nine other fungi from five different families under the order Hypocreales, provide confirmation that *P. lilacinum*, which was earlier erroneously grouped with *Paecilomyces* under the order Eurotiales, belongs to the Ophiocordycipitaceae family in the order Hypocreales. In conclusion, findings of this genomic analysis would be beneficial for development of effective biological-control approaches to manage agricultural damage incurred by plant parasitic nematodes.

## Methods

### Fungal strain

The details of *P. lilacinum* strain used in the study has been studied in the laboratory for more than 7 years [56]. *P. lilacinum* grown on potato dextrose agar (PDA) at 25 °C for 8 days was inoculated in potato dextrose broth (PDB) and incubated at 25 °C for 5 days with shaking at 120 rpm. Genomic DNA was isolated using the DNeasy Plant Maxi Kit (Qiagen) following the fungal DNA isolation protocol. Total DNA obtained was subjected to quality control by agarose gel electrophoresis and quantified by the nanodrop method.

### Genome sequencing and assembly

Genomic sequencing was performed at MOgene LC, USA, using next generation sequencing technology Illumina. Two paired end libraries (insert sizes 180 bp and 500 bp) and one mate pair library (5Kb) were constructed. Briefly, for paired end libraries, 1 μg genomic DNA in total volume of 50 μL was fragmented using Covaris to generate dsDNA fragments with 3’ or 5’ overhangs. After shearing, the ends of DNA fragments were repaired by adding 40 μL of Illumina end repair mix and incubation at 30 °C for 30 min, followed by adenylation of 3’ ends (added 2.5uL of A-Tailing mix and incubated at 37 °C for 30 min). Double-stranded Illumina paired-end adapters were ligated to the polyadenylated DNA. Ligated product was purified by loading 20 μL of samples on 1 % agarose gel. For 180 bp insert size, bands from the gel ranging from 300-400 bp were excised and for 500 bp insert size, bands ranging between 620-700 bp were excised. Excised bands were then purified using QIA gel extraction kit. 20 μL of purified sample were PCR amplified using the following program (98 °C for 30 s; 10 cycles of: 98 °C for 10 s, 60 °C for 30 s, 72 °C for 30 s; 72 °C for 5 min). DNA libraries were purified using AMPure XP beads. KAPA was done to quantify the libraries, which were then normalized and pooled at 4nM concentration.

For constructing mate pair library of insert size ~ 5 Kb, 4 μg of gDNA was tagmented using mate pair tagment enzyme followed by strand displacement at 20 °C for 30 min. AMPure beads were used to purify strand displaced samples. Smaller fragments of size less than 1500 bp were removed. 30 μL of samples were run on 1 % agarose gel and bands ranging from 5-7 kb were excised and cleaned using Zymoclean large fragment DNA recovery kit. Purified fragments were circularized with an overnight incubation at 30 °C followed by DNA exonuclease treatment to remove any linear molecules still remaining in the circularization reaction. Circularized DNA molecules were then sheared to small sized fragments (approximately 300-1000 bp). After shearing, the ends of DNA fragments were repaired, ligated with appropriate adaptor, PCR amplified, and purified in the manner described above for paired end library preparation.

Libraries were quantified and loaded into Illumina flow-cells at concentrations of 1.4–1.75 pM (HiSeq 2000). Cluster generation was performed in a cBOT automated cluster generation system. Base calling was done using Illumina pipeline software. A total of 5.4 Gb of pair-end (180 bp and 500 bp inserts) and 2.6 Gb of mate-pairs (5 kb inserts), were produced. A total of 8 Gb raw data was subjected to adaptor- and quality-based trimming. Quality-passed data was assembled using the *de novo* genome assembler AllpathsLG [57]. Reads with overlaps were first combined to form contigs. The reads were mapped back to contigs. With paired-end reads, contigs from the same transcript, as well as the distances between these contigs, were detected. In order to generate scaffolds, contigs were connected using “N” to represent unknown sequences between two contigs. Mate-pair reads were used for gap filling of scaffolds in order to get sequences with minimal N's and the longest length. By mapping the reads to contigs, the genome sequence was assembled into 301 scaffolds with total gap size of 526,913 bases. The whole genome project has been deposited at https://submit.ncbi.nlm.nih.gov/subs/wgs/ under Bioproject number PRJNA284314.

### Gene prediction, annotation, protein classification and phylogenomic analysis

#### Genomic structural annotation

Maker [25] was used to structurally annotate the genomic assembly. EST data for 21 different fungal species, and protein sequences from 10 different fungal species were used to inform the annotation processes. SNAP [58], GeneMarkHMM [59], and Augustus [60], each trained on fungal data and informed with EST and protein sequences as described above, were used to predict genes.

### Functional annotation

Repeat elements in the genome were analyzed by RMBlastN search against the RepeatMasker library (open 3.2.9) [61]. The DNA transposases and reterotransposaes were annotated by blastp searches against Repbase (repeatmaskerlibraries-20140131) [62]. To estimate genome size k-mer analysis was carried out using KmerGenie v1.6976 [63]. Pseudogene identification was conducted using the PseudoPipe tool [64]. tRNAs were identified using tRNA scan-SE [28, 29]. Protein set derived from genomic structural annotation was annotated using Blastp versus the NCBI/Genbank NR protein database [65], and using the protein domain/motif prediction program InterProScan [66]. Genes were also annotated by using Blast2GO [67] based on the terms “biological function” and “molecular process” in Gene Ontology (GO). SignalP [68] was used to identify putative secreted proteins. Proteins coding for proteases were classified by conducting Blastp (batch) against the MEROPS database [46, 47]. In order to carry out classification of carbohydrate-active enzymes (CAZymes), a HMMER scan was carried out based on the profiles compiled in dbCAN release 2.0 on the CAZy database. To identify G-protein-coupled receptors in the genome, local BLAST to GPCRDB sequences [69] was carried out and those proteins containing seven trans-membrane helices were selected using TMHMM [70]. Homologs of the Pth11-like GPCRs were confirmed by local Blastp analysis (E value of 1e-20). Secondary metabolite genes were predicted by SMURF [54]. AntiSMASH [55] analysis on the whole genome sequence was performed to identify classical and novel clusters coding for secondary metabolites. Blastp search (E value < 1*10^−20^) against pathogen-host interaction (PHI) database ver 3.6 [35] was carried out to identify putative pathogenesis genes.

### Phylogenomic analysis

Whole genome nucleotide, gene and protein sequences of nine species belonging to the fungal order Hypocreales namely, *Fusarium oxysporum (**http://www.ncbi.nlm.nih.gov/bioproject/18813**), Fusarium graminearum* (http://www.ncbi.nlm.nih.gov/bioproject/PRJNA235346)*, Trichoderma reesei* (http://www.ncbi.nlm.nih.gov/bioproject/PRJNA266930)*, Beauveria bassiana* (http://www.ncbi.nlm.nih.gov/bioproject/38719)*, Cordyceps militaris (*http://www.ncbi.nlm.nih.gov/bioproject/PRJNA41129/)*, Metarhizium robertsii (*http://www.ncbi.nlm.nih.gov/bioproject/PRJNA245140/)*, Metarhizium acridum* (http://www.ncbi.nlm.nih.gov/bioproject/38715/)*, Pochonia chlamydosporia (*http://www.ncbi.nlm.nih.gov/bioproject/68669/) and *Tolypocladium inflatum (*http://www.ncbi.nlm.nih.gov/bioproject/PRJNA73163/), were downloaded from NCBI database (http://www.ncbi.nlm.nih.gov/). In the absence of gene/protein sequences of *Pochonia chlamydosporia* and *Tolypocladium inflatum*, structural annotation (prediction of gene and protein sequences) of their respective BioProjects present in NCBI was carried out using Augustus [60]. To serve as an out group in the phylogenomic analysis, protein sequence of *Neurospora crassa* was downloaded (http://www.broadinstitute.org/annotation/genome/neurospora/). Hal, an automated pipeline for phylogenetic analysis of genome-scale data [31] was used for multiple alignment and generation of orthologous protein clusters [http://sourceforge.net/projects/bio-hal/]. Orthologous protein clusters [71] were identified by using Blastp and Markov Cluster Algorithm (MCL) tool [72] integrated in Hal pipeline. Only non-redundant clusters that consisted of protein sequences with best hits to proteins from own cluster were filtered out. Protein sequences for these orthologous clusters were extracted from proteome dataset and aligned using MUSCLE [73]. Gblocks was used to remove poorly aligned positions and highly divergent sequences [74]. Individual alignments were parsed by minimum alignment length and concatenated using FasConcat [75] for generation of super alignments. RAxML [76] with PROTCAT setting, Dayhoff amino acid substitution model and 1000 bootstrap replications was run on super-aligned sequences for construction of phylogenetic tree.

### Quantitative real-time PCR

#### Total RNA extraction and cDNA preparation

The *P. lilacinum* strain used in the study was grown on potato dextrose media in the presence of the nematode *Meloidogyne incognita* eggs. Same strain grown in the absence of *Meloidogyne incognita* eggs served as control. The cultures were incubated for 0, 4, 7 and 10 day/s before RNA isolation. Total RNA was extracted using PureLink RNA Mini Kit (Invitrogen, USA). cDNA for all the samples corresponding to different time points (0, 4, 7 and 10 day/s) was prepared using iScript cDNA synthesis kit (Bio-Rad Laboratories, Inc.) using following incubation conditions: 5 min at 25 °C, 30 min at 42 °C and finally 5 min at 85 °C. Quantification of RNA and cDNA samples was done using the H1M microplate reader (Biotek, USA).

### Primer designing and real-time PCR analysis

To facilitate the real-time PCR analysis of the three serine protease genes and reference actin gene under same reaction conditions, primers were designed using Primer Express 3.0 software (PE Applied Biosystems, USA) under default parameters. The forward and reverse primers, wherever possible, were designed spanning an intron to detect any genomic DNA contamination. The primer sequences are given in Additional file 1: Table S17.

### Gene expression and relative quantification

Quantification of gene expression was performed on CFX- 96 real-time PCR detection system (Bio-Rad Labs, Inc.). The PCR mixture contained 100 ng of total cDNA template, 10 μl of 2X Sso Fast Eva Green Supermix Dye (Bio-Rad Labs, Inc.), 100 nM forward and reverse primer in a final volume of 20 μl. Thermal cycling conditions were as follows: 30 s at 95 °C followed by 40 repeats of 10 s at 95 °C, 30 s at 60 °C and a melt curve from 65 °C to 95 °C. Data collection was performed during each cycle. Relative gene expression by qPCR was performed using Actin as the reference gene for normalization of expression of target genes. PCRs with no template controls (NTC) were also performed for each primer pair. The specificity of amplicons was verified by melting curve analysis (60 °C to 95 °C) after 40 cycles. Two biological replicates for each sample were used for real-time PCR analysis and three technical replicates were analyzed for each biological replicate.

### Statistical analyses

Data for qPCR reactions were reported with standard error from the mean. The data was analyzed using the nonparametric Kruskale-Wallis test. These were then subjected to pairwise comparison. The significance level used for analysis was *P* < 0.05 for all statistical tests. Statistical analyses were performed using SPSS version 12.0.

## Additional file

Additional file 1:
**Table S1.** Statistics of the completeness of the genome based on 248 CEGs. **Table S2.** Genome size and the number of protein-coding genes in *P. lilacinum* and the other Hypocreales fungi. **Table S3.** Repeat elements in the draft genome sequence of *P. lilacinum.* **Table S4.** Total number of genes sharing homology with PHI db in the 10 Hypocreales genomes. **Table S5.** Fisher’s exact test for evaluation of difference in genetic make-up between *P. lilacinum* and *P. chlamydosporia.* **Table S6.** Gene coding for secreted proteins sharing homology with PHI db. **Table S7.** GPCRs sharing homology with Pth11-like GPCRs in *P. lilacinum.* **Table S8.** Genes coding for GPCRs, Histidine kinases, protein kinases, ABC transporters, MFS and CYP450 proteins in *P. lilacinum.* **Table S9.** Genes coding for proteases in the *P. lilacinum* genome. **Table S10.** Gene coding for serine proteases with homologs in the PHI db. **Table S11.** Carbohydrate-degrading enzymes arranged by GH family. **Table S12.** Carbohydrate esterases arranged by CE family. **Table S13.** Glycosides hydrolase and carbohydrate esterase genes sharing homology with PHI db. **Table S14.** Secondary metabolites genes and clusters identified by SMURF. **Table S15.** AntiSMASH based functional annotation of secondary metabolite clusters. **Table S16.** NaPDoS analysis to detect condensation (C) and ketosynthase (KS) domains. **TableS 17.** Primer sequences for quantitative real-time PCR analyses. **Figure S1.** k-mer analysis to predict assembly size of *P. lilacinum*. (ZIP 201 kb)

## Abbreviations

*P. lilacinum*: *Purpureocillium lilacinum*
PHI: Pathogen–host interaction
CAZy: Carbohydrate-active enzyme
GH: Glycoside hydrolase
CE: Carbohydrate esterase
PKS: Polyketide synthase
NRPS: Non-ribosomal peptide synthetase
MFS: Major facilitator superfamily
ABC: ATP-binding cassette
CYP: Cytochrome P450
GPCR: G-protein coupled receptor
