# Supplementary material for: Whole genome annotation and comparative genomic analyses of bio-control fungus Purpureocillium lilacinum
Source: BMC Genomics. 2015 Nov 25;16:1004. doi: 10.1186/s12864-015-2229-2 (PMC4658809; doi:10.1186/s12864-015-2229-2)
Supplement: Additional file 1: — Table S1. Statistics of the completeness of the genome based on 248 CEGs. Table S2. Genome size and the number of protein-coding genes in P. lilacinum and the other Hypocreales fungi. Table S3. Repeat elements in the draft genome sequence of P. lilacinum. Table S4. Total number of genes sharing homology with PHI db in the 10 Hypocreales genomes. Table S5. Fisher’s exact test for evaluation of difference in genetic make-up between P. lilacinum and P. chlamydosporia. Table S6. Gene coding for secreted proteins sharing homology with PHI db. Table S7. GPCRs sharing homology with Pth11-like GPCRs in P. lilacinum. Table S8. Genes coding for GPCRs, Histidine kinases, protein kinases, ABC transporters, MFS and CYP450 proteins in P. lilacinum. Table S9. Genes coding for proteases in the P. lilacinum genome. Table S10. Gene coding for serine proteases with homologs in the PHI db. Table S11. Carbohydrate-degrading enzymes arranged by GH family. Table S12. Carbohydrate esterases arranged by CE family. Table S13. Glycosides hydrolase and carbohydrate esterase genes sharing homology with PHI db. Table S14. Secondary metabolites genes and clusters identified by SMURF. Table S15. AntiSMASH based functional annotation of secondary metabolite clusters. Table S16. NaPDoS analysis to detect condensation (C) and ketosynthase (KS) domains. TableS 17. Primer sequences for quantitative real-time PCR analyses. Figure S1. k-mer analysis to predict assembly size of P. lilacinum. (ZIP 201 kb) [file 12864_2015_2229_MOESM1_ESM.zip › additional file 1/additional_file1_15-sept.docx]

**Additional Files**

**Additional File: Table S1**: Statistics of the completeness of the genome based on 248 CEGs

|  | #Prots | %Completeness | - | #Total | Average | %Ortho |
| --- | --- | --- | --- | --- | --- | --- |
| Complete | 240 | 96.77 | - | 268 | 1.12 | 8.75 |
| Group1 | 63 | 95.45 | - | 73 | 1.16 | 11.11 |
| Group2 | 52 | 92.86 | - | 54 | 1.04 | 3.85 |
| Group3 | 61 | 100 | - | 72 | 1.18 | 14.75 |
| Group4 | 64 | 98.46 | - | 69 | 1.08 | 4.69 |
|  |  |  |  |  |  |  |
| Partial | 243 | 97.98 | - | 280 | 1.15 | 11.52 |
| Group1 | 65 | 98.48 | - | 75 | 1.15 | 10.77 |
| Group2 | 53 | 94.64 | - | 58 | 1.09 | 7.55 |
| Group3 | 61 | 100 | - | 76 | 1.25 | 19.67 |
| Group4 | 64 | 98.46 | - | 71 | 1.11 | 7.81 |

# Key:

# Prots = number of 248 ultra-conserved CEGs present in genome

# %Completeness = percentage of 248 ultra-conserved CEGs present

# Total = total number of CEGs present including putative orthologs

# Average = average number of orthologs per CEG

# %Ortho = percentage of detected CEGS that have more than 1 ortholog

**Additional File 1:**  **Table S2:** Genome size and the number of protein-coding genes in *P. lilacinum* and the other Hypocreales fungi

| **Species** | **Genome size (Mb)** | **No. of genes** | **NCBI accession** | **Release Date** |
| --- | --- | --- | --- | --- |
| *F. oxysporum* | 61.3 | 27347 | [AAXH01](http://www.ncbi.nlm.nih.gov/Traces/wgs/?val=AAXH01)00000000 | 2010/12/27 |
| *F. graminearum* | 36.6 | 13321 | [JATU01](http://www.ncbi.nlm.nih.gov/Traces/wgs/?val=JATU01)00000000 | 2014/03/25 |
| *T. reesei* | 33.3 | 9115 | [AAIL00000000.2](http://www.ncbi.nlm.nih.gov/nuccore/AAIL00000000.2) | 2014/08/01 |
| *B.bassiana* | 33.6 | [10364](http://www.ncbi.nlm.nih.gov/genome/proteins/910?genome_assembly_id=31910) | [ADAH01](http://www.ncbi.nlm.nih.gov/Traces/wgs/?val=ADAH01) | 2014/08/11 |
| *C. militaris* | 32.2 | [9651](http://www.ncbi.nlm.nih.gov/genome/proteins/10687?genome_assembly_id=39585) | [AEVU01](http://www.ncbi.nlm.nih.gov/Traces/wgs/?val=AEVU01) | 2014/08/11 |
| *M. acridum* | 38.0 | 9,849 | ADNI01000000 | 2014/08/11 |
| *M. robertsii* | 41.65 | 11,688 | [ADNJ00000000](http://www.ncbi.nlm.nih.gov/nuccore/ADNJ00000000).2 | 2014/12/19 |
| *P.* *chlamydosporia** | 41.0 | 10,124 (predicted by Augustus) | [AOSW00000000](http://www.ncbi.nlm.nih.gov/nuccore/AOSW00000000) | 2013/06/17 |
| *P. lilacinum* | 40.02 | 13266 |  | - |
| *T. inflatum** | 30.22 | 10774 | [AOHE00000000.1](http://www.ncbi.nlm.nih.gov/nuccore/523397120) | 2013/07/10 |

*Structural annotation done by Augustus in this study

**Additional File:Table S3:** Repeat Elements in the draft genome sequence of *P. lilacinum*

RepeatMasker

**==================================================**

Genome Name: *P. lilacinum* (assembly)

Sequences: 301; Total length: 40017242 bp (39491088 bp excl N/X-runs);

GC level: 58.57 %

Bases masked: 672777 bp (1.68 %)

|  | Number of Elements* | Length occupied | Percentage of Sequences |
| --- | --- | --- | --- |
| Retroelements | 105 | 25023 bp | 0.06% |
| SINEs: | 0 | 0 bp | 0% |
| Penelope | 0 | 0 bp | 0% |
| LINEs: | 71 | 15521 bp | 0.04% |
| CRE/SLACS | 0 | 0 bp | 0% |
| L2/CR1/Rex | 0 | 0 bp | 0% |
| R1/LOA/Jockey | 0 | 0 bp | 0% |
| R2/R4/NeSL | 0 | 0 bp | 0% |
| RTE/Bov-B | 0 | 0 bp | 0% |
| L1/CIN4 | 0 | 0 bp | 0% |
| LTR elements: | 34 | 9502 bp | 0.02% |
| BEL/Pao | 0 | 0 bp | 0% |
| Ty1/Copia | 24 | 5253 bp | 0.01% |
| Gypsy/DIRS1 | 10 | 4249 bp | 0.01% |
| Retroviral | 0 | 0 bp | 0% |
|  |  |  |  |
| DNA transposons | 2 | 193 bp | 0% |
| hobo-Activator | 0 | 0 bp | 0% |
| Tc1-IS630-Pogo | 2 | 193 bp | 0% |
| En-Spm | 0 | 0 bp | 0% |
| MuDR-IS905 | 0 | 0 bp | 0% |
| PiggyBac | 0 | 0 bp | 0% |
| Tourist/Harbinger | 0 | 0 bp | 0% |
| Other (Mirage, | 0 | 0 bp | 0% |
| P-element, Transib) |  |  |  |
| Rolling-circles | 0 | 0 bp | 0% |
|  |  |  |  |
| Unclassified: | 1 | 67 bp | 0% |
| Total interspersed repeats: |  | 25283 bp | 0.06% |
|  |  |  |  |
| Small RNA: | 1 | 66 bp | 0% |
| Satellites: | 26 | 3457 bp | 0.01% |
| Simple repeats: | 14386 | 595034 bp | 1.49% |
| Low complexity: | 998 | 49079 bp | 0.12% |

=================================================

**Additional File 3: Table S4:** Total number of genes sharing homology with PHI db in the 10 Hypocreales genomes

| Query | Database | BLAST | **No. of genes (E<1*10^-20^)** |
| --- | --- | --- | --- |
| *F. oxysporum* | PHI Database | BLASTP | 2486 |
| *F. graminearum* | PHI Database | BLASTP | 1952 |
| *T. reesei* | PHI Database | BLASTP | 1409 |
| *B. bassiana* | PHI Database | BLASTP | 1507 |
| *C. militaris* | PHI Database | BLASTP | 1509 |
| *P. chalamadosporia* | PHI Database | BLASTP | 1402 |
| *M. acridum* | PHI Database | BLASTP | 1477 |
| *M. robertsii* | PHI Database | BLASTP | 1593 |
| *P .lilacinum* | PHI Database | BLASTP | 1953 |
| *T. inflatum* | PHI Database | BLASTP | 1470 |

| **genes** | ***P. lilacinum* (n=13266)** | ***P. chlamydosporia***  **(n=10124)** | **P** |
| --- | --- | --- | --- |
| phi genes | 1953 | 1402 | 0.05896 |
| establishment of localization | 959 | 1141 | 0 |
| primary metabolic process | 2291 | 2182 | 0 |
| nitrogen compound metabolic process | 1288 | 719 | 0 |
| protein binding | 970 | 1051 | 0 |
| Glycoside hydrolases | 249 | 281 | 5E-06 |
| Sec.metabolites | 30 | 42 | 0.00984 |
| response to stress | 159 | 199 | 0.009877 |
| serine proteases | 209 | 189 | 0.087764 |
| Carbohydrate esterase | 106 | 95 | 0.252691 |

**Table S5:** Fisher’s exact test for evaluation of difference in genetic make-up between *P. lilacinum* and *P. chlamydosporia*

**Additional file 1: Table S9:** Genes coding for proteases in the *P. lilacinum* genome

| **Hydrolytic Enzyme type** | **Catalytic Type** | **Family** | **Description** | **Number of Genes** | | |
| --- | --- | --- | --- | --- | --- | --- |
|  |  |  |  | *M.acridum* | *M. robertsii* | *P. lilacinum* |
| **Protease** | **Aspartic Proteases** | A01 | Acid proteinases | 24 | 31 | 28 |
|  |  | A22 | Membrane-inserted  Endopeptidases | 3 | 1 | 1 |
|  |  | A28 | DNA-damage-inducible proteins | 1 | 1 | 1 |
|  |  | A33 | skinSASPase | 1 | 1 | 1 |
|  | **Cysteine Proteases** | C01 | Endopeptidases/ papains | 1 | 2 | 2 |
|  |  | C02 | Endopeptidases /calpains | 1 | 1 | 3 |
|  |  | C12 | Ubiquitinyl hydrolases | 5 | 4 | 5 |
|  |  | C13 | Legumain | 1 | 1 | 1 |
|  |  | C14 | Cytosolic endopeptidases | 2 | 2 | 2 |
|  |  | C15 | pyroglutamyl-peptidase I | 0 | 1 | 1 |
|  |  | C19 | Ubiquitinyl hydrolases | 26 | 17 | 14 |
|  |  | C26 | peptidasew-glutamyl hydrolases | 13 | 11 | 12 |
|  |  | C40 | Dipeptidyl-peptidases | 1 | 1 | 2 |
|  |  | C44 | Amidophosphoribosyltransferases precursor | 6 | 5 | 4 |
|  |  | C45 | Autolytic endopeptidase | 0 | 1 | 1 |
|  |  | C48 | small ubiquitin-like modifier | 4 | 4 | 4 |
|  |  | C50 | Endopeptidases/separases | 1 | 1 | 1 |
|  |  | C54 | Endopeptidase of glycyl bonds | 1 | 1 | 1 |
|  |  | C56 | Putative PfpI endopeptidases | 8 | 8 | 8 |
|  |  | C65 | Isopeptidase | 1 | 1 | 1 |
|  |  | C85 | Deubiquitinylating peptidases | 2 | 2 | 2 |
|  |  | C97 | DeSI-1peptidase | 2 | 2 | 2 |
|  |  | G01 | scytalidoglutamic peptidase | 1 | 4 | 6 |
|  |  | I01 | Ovomucoid | 0 | 2 |  |
|  |  | I02 | Aprotinin | 1 | 0 | 1 |
|  |  | I07 | trypsin inhibitor | 0 | 1 | 0 |
|  |  | I08 | chymotrypsin/elastase inhibitor | 0 | 2 | 0 |
|  |  | I09 | peptidase B inhibitor | 1 | 4 | 8 |
|  |  | I20 | potato peptidase inhibitor | 0 | 0 | 2 |
|  |  | I32 | Surviving | 0 | 1 | 2 |
|  |  | I51 | serine carboxypeptidase Y inhibitor | 0 | 0 | 1 |
|  |  | I78 | elastase inhibitor | 0 | 0 | 1 |
|  |  | I87 | HflC protein | 2 | 2 | 2 |
|  | **Metalloproteases** | M01 | Zinc aminopeptidases | 5 | 4 | 4 |
|  |  | M03 | Oligopeptidases | 8 | 5 | 7 |
|  |  | M04 | Zinc metalloproteases | 1 | 1 | 2 |
|  |  | M06 | Zinc Metalloproteases | 0 | 0 | 1 |
|  |  | M10 | Zinc metalloendopeptidases | 0 | 3 | 2 |
|  |  | M12 | 'ADAM'peptidases | 3 | 2 | 2 |
|  |  | M14 | Metallocarboxypeptidases | 4 | 5 | 3 |
|  |  | M16 | Metalloendopeptidases | 10 | 8 | 7 |
|  |  | M18 | Metalloaminopeptidases | 2 | 2 | 2 |
|  |  | M19 | Dipeptidases | 0 | 1 | 2 |
|  |  | M20 | Glutamate carboxypeptidases | 8 | 9 | 18 |
|  |  | M24 | Methionyl aminopeptidases | 11 | 11 | 12 |
|  |  | M28 | Aminopeptidases andcarboxypeptidases | 11 | 9 | 11 |
|  |  | M35 | Deuterolysins | 7 | 7 | 7 |
|  |  | M36 | Fungalysins | 2 | 2 | 2 |
|  |  | M38 | Isoaspartyl dipeptidase | 12 | 12 | 14 |
|  |  | M41 | ATP-dependent metalloendopeptidases | 2 | 2 | 2 |
|  |  | M43 | Cytophagalysins | 6 | 19 | 3 |
|  |  | M48 | Mating proteases | 2 | 2 | 2 |
|  |  | M49 | Dipeptidylpeptidases | 2 | 2 | 2 |
|  |  | M67 | PSMD14 peptidases | 7 | 7 | 7 |
|  |  | M76 | Atp23 peptidase | 0 | 1 | 1 |
|  |  | M77 | tryptophanyl aminopeptida | 5 | 6 | 1 |
|  |  | M79 | RCE1peptidase | 1 | 0 | 1 |
|  |  | M80 | Wss1peptidase | 3 | 3 | 3 |
|  |  | M81 | microcystinase MlrC | 1 | 1 | 1 |
|  | **Mixed proteases** | P01 | DmpAaminopeptidase | 2 | 3 | 2 |
|  | **Serine proteases** | S01 | Trypsins | 16 | 37 | 4 |
|  |  | S08 | Subtilisins | 43 | 49 | 35 |
|  |  | S09 | Prolyl oligopeptidases | 41 | 56 | 69 |
|  |  | S10 | Serine carboxypeptidases | 14 | 11 | 11 |
|  |  | S12 | Ala-D-Ala carboxypeptidases | 18 | 17 | 23 |
|  |  | S14 | Peptidase Clp (type 1) | 1 | 1 | 1 |
|  |  | S16 | P-dependent peptidases | 3 | 3 | 2 |
|  |  | S26 | Signal peptidases | 3 | 3 | 2 |
|  |  | S28 | Exopeptidases of prolyl bonds | 6 | 6 | 2 |
|  |  | S33 | Prolyl aminopeptidases | 46 | 43 | 53 |
|  |  | S53 | Sedolisin | 11 | 9 | 9 |
|  |  | S54 | Rhomboid | 2 | 2 | 3 |
|  |  | S59 | Autolytic endopeptidases | 1 | 1 | 1 |
|  | **Threonine**  **Proteases** | T01 | Proteasome and related compound Peptidases | 16 | 14 | 14 |
|  |  | T02 | N-terminal nucleophile hydrolases | 6 | 4 | 3 |
|  |  | T03 | Aminopeptidases | 4 | 5 | 6 |
|  |  | T05 | Ornithine acetyltransferases | 1 | 1 | 1 |

**Additional File:** **Table S11:** Carbohydrate-degrading enzymes arranged by GH family

| **GH family** | ***F. graminearum*** | ***M. acridum*** | ***M. robertsii*** | ***P. chlamydosporia*** | ***P. lilacinum*** |
| --- | --- | --- | --- | --- | --- |
| GH1 | 3 | 3 | 3 | 3 | 3 |
| GH2 | 10 | 6 | 6 | 11 | 12 |
| GH3 | 22 | 7 | 9 | 16 | 11 |
| GH4 | 1 | 0 | 0 | 0 | 0 |
| GH5 | 14 | 9 | 8 | 11 | 11 |
| GH6 | 1 | 0 | 0 | 1 | 0 |
| GH7 | 2 | 0 | 0 | 1 | 0 |
| GH10 | 5 | 0 | 0 | 1 | 0 |
| GH11 | 2 | 0 | 0 | 1 | 0 |
| GH12 | 4 | 1 | 1 | 1 | 2 |
| GH13 | 7 | 5 | 5 | 7 | 8 |
| GH15 | 3 | 2 | 2 | 2 | 3 |
| GH16 | 24 | 18 | 19 | 18 | 19 |
| GH17 | 4 | 4 | 5 | 5 | 4 |
| GH18 | 18 | 19 | 28 | 19 | 28 |
| GH20 | 3 | 2 | 2 | 9 | 7 |
| GH23 | 1 | 1 | 1 | 1 | 1 |
| GH24 | 0 | 2 | 2 | 4 | 4 |
| GH25 | 0 | 2 | 2 | 4 | 2 |
| GH27 | 2 | 2 | 2 | 2 | 2 |
| GH28 | 6 | 1 | 1 | 4 | 3 |
| GH29 | 1 | 2 | 1 | 3 | 3 |
| GH30 | 0 | 0 | 0 | 2 | 1 |
| GH31 | 8 | 6 | 6 | 8 | 6 |
| GH32 | 6 | 2 | 2 | 3 | 3 |
| GH33 | 1 | 1 | 1 | 1 | 1 |
| GH35 | 3 | 3 | 3 | 3 | 2 |
| GH36 | 2 | 1 | 1 | 2 | 1 |
| GH37 | 3 | 2 | 2 | 2 | 2 |
| GH38 | 1 | 1 | 1 | 1 | 1 |
| GH39 | 0 | 0 | 0 | 0 | 0 |
| GH43 | 19 | 3 | 1 | 11 | 4 |
| GH45 | 1 | 0 | 0 | 1 | 1 |
| GH47 | 10 | 9 | 8 | 11 | 7 |
| GH49 | 0 | 0 | 0 | 1 | 1 |
| GH51 | 2 | 0 | 0 | 0 | 1 |
| GH53 | 1 | 0 | 0 | 0 | 0 |
| GH54 | 1 | 0 | 1 | 3 | 3 |
| GH55 | 3 | 4 | 5 | 8 | 8 |
| GH62 | 1 | 0 | 0 | 0 | 0 |
| GH63 | 1 | 1 | 1 | 1 | 1 |
| GH64 | 2 | 1 | 1 | 3 | 2 |
| GH65 | 0 | 1 | 1 | 1 | 1 |
| GH67 | 1 | 0 | 0 | 1 | 0 |
| GH71 | 0 | 1 | 1 | 0 | 1 |
| GH72 | 3 | 5 | 5 | 0 | 5 |
| GH73 | 1 | 0 | 0 | 0 | 0 |
| GH74 | 5 | 4 | 3 | 0 | 1 |
| GH75 | 1 | 3 | 3 | 0 | 4 |
| GH76 | 8 | 13 | 15 | 0 | 14 |
| GH78 | 7 | 0 | 0 | 0 | 1 |
| GH79 | 1 | 1 | 1 | 0 | 6 |
| GH81 | 1 | 2 | 1 | 2 | 1 |
| GH84 | 0 | 1 | 1 | 0 | 1 |
| GH88 | 1 | 0 | 1 | 3 | 1 |
| GH89 | 0 | 1 | 2 | 2 | 1 |
| GH92 | 0 | 6 | 5 | 4 | 5 |
| GH93 | 2 | 0 | 0 | 1 | 1 |
| GH95 | 3 | 0 | 1 | 4 | 3 |
| GH99 | 0 | 0 | 1 | 0 | 0 |
| GH105 | 3 | 1 | 3 | 3 | 4 |
| GH106 | 1 | 0 | 0 | 0 | 0 |
| GH109 | 15 | 8 | 6 | 10 | 11 |
| GH114 | 2 | 1 | 1 | 2 | 1 |
| GH115 | 3 | 1 | 1 | 1 | 2 |
| GH117 | 0 | 0 | 1 | 0 | 0 |
| GH121 | 1 | 0 | 0 | 0 | 1 |
| GH125 | 3 | 3 | 3 | 2 | 4 |
| GH127 | 2 | 1 | 1 | 1 | 2 |
| GH128 | 4 | 3 | 4 | 3 | 6 |
| GH130 | 0 | 0 | 0 | 0 | 1 |
| GH131 | 1 | 0 | 0 | 0 | 0 |
| GH132 | 2 | 2 | 2 | 2 | 2 |
| GH133 | 0 | 0 | 1 | 0 | 0 |
| TOTAL | 269 | 176 | 194 | 281 | 249 |

**Additional File: Table S12:** Carbohydrate esterases arranged by CE family

|  | **Hypocreales** | | | | | **Magnaporthales** |
| --- | --- | --- | --- | --- | --- | --- |
| **CE Family** | **F. *graminearum*** | ***M. acridum*** | ***M. robertsii*** | ***P. chlamydosporia*** | ***P. lilacinum*** | ***N. crassa*** |
| CE1 | 29 | 20 | 20 | 25 | 22 | 35 |
| CE2 | 1 | 0 | 0 | 0 | 0 | 2 |
| CE3 | 7 | 2 | 5 | 3 | 4 | 8 |
| CE4 | 9 | 3 | 6 | 3 | 8 | 12 |
| CE5 | 13 | 2 | 2 | 5 | 10 | 18 |
| CE7 | 1 | 0 | 1 | 0 | 1 | 0 |
| CE8 | 7 | 0 | 0 | 0 | 0 | 1 |
| CE9 | 1 | 1 | 1 | 1 | 1 | 1 |
| CE10 | 66 | 34 | 41 | 55 | 57 | 50 |
| CE12 | 5 | 2 | 0 | 0 | 0 | 3 |
| CE14 | 0 | 2 | 2 | 2 | 2 | 1 |
| CE15 | 0 | 0 | 0 | 0 | 0 | 1 |
| CE16 | 5 | 1 | 2 | 1 | 1 | 2 |
| TOTAL | 144 | 67 | 80 | 95 | 106 | 135 |

**Additional File:TableS14:** The number of Secondary metabolites genes and clusters identified by SMURF

| **Pathway** | **FO** | **FG** | **CM** | **PC** | **MAC** | **MR** | **PL** | **TI** |
| --- | --- | --- | --- | --- | --- | --- | --- | --- |
| DMAT | 2 | 0 | 0 | 0 | 3 | 6 | 1 | 1 |
| HYBRID | 2 | 1 | 3 | 4 | 1 | 5 | 1 | 4 |
| NRPS | 7 | 10 | 5 | 12 | 13 | 15 | 7 | 14 |
| NRPS-like | 12 | 11 | 8 | 8 | 8 | 14 | 7 | 11 |
| PKS | 9 | 14 | 9 | 15 | 13 | 25 | 12 | 20 |
| PKS-like | 2 | 1 | 2 | 3 | 4 | 3 | 2 | 5 |
| Total | 36 | 37 | 27 | 42 | 39 | 68 | 30 | 55 |

**Note: DMAT, Dimethylallyl tryptophan *synthase;* NRPS, non-ribosomal peptide synthetases; PKS, polyketide synthetases; HYBRID, hybrid PKS–NRPS enzyme. The species: FO, *Fusarium oxysporum*;**

**Additional file 1: TableS17:** Primer sequences for quantitative real-time PCR analyses

| AC_g3526.t1_ F | 5’TGACCGAGTTCAAGGAGTCC3’ |
| --- | --- |
| AC_g3526.t1_R | 5’GTCGGGCATCTCAAAGACAC3’ |
| SePro_g3158.t1_F | 5’GCGGCTACATCTTTGAGGTG3’ |
| SePro_g3158.t1_R | 5’AGTTGAGCTTCATTCGCGTC3’ |
| SePro_g3207.t1_F | 5’GCTCTAGGCGGTTGTTTCG3’ |
| SePro_ g3207.t1_R | 5’CAGCGCCAGTGAAGTGTAG3’ |
| SePro_g10487.t1_F | 5’TTGGCAAGAAGGAGATCACC3’ |
| SePro_g10487.t1_R | 5’CAGTCGCTTGCCATctGTC3’ |
