# Supplementary material for: Whole genome annotation and comparative genomic analyses of bio-control fungus Purpureocillium lilacinum
Source: BMC Genomics. 2015 Nov 25;16:1004. doi: 10.1186/s12864-015-2229-2 (PMC4658809; doi:10.1186/s12864-015-2229-2)
Supplement: Additional file 1: — Table S1. Statistics of the completeness of the genome based on 248 CEGs. Table S2. Genome size and the number of protein-coding genes in P. lilacinum and the other Hypocreales fungi. Table S3. Repeat elements in the draft genome sequence of P. lilacinum. Table S4. Total number of genes sharing homology with PHI db in the 10 Hypocreales genomes. Table S5. Fisher’s exact test for evaluation of difference in genetic make-up between P. lilacinum and P. chlamydosporia. Table S6. Gene coding for secreted proteins sharing homology with PHI db. Table S7. GPCRs sharing homology with Pth11-like GPCRs in P. lilacinum. Table S8. Genes coding for GPCRs, Histidine kinases, protein kinases, ABC transporters, MFS and CYP450 proteins in P. lilacinum. Table S9. Genes coding for proteases in the P. lilacinum genome. Table S10. Gene coding for serine proteases with homologs in the PHI db. Table S11. Carbohydrate-degrading enzymes arranged by GH family. Table S12. Carbohydrate esterases arranged by CE family. Table S13. Glycosides hydrolase and carbohydrate esterase genes sharing homology with PHI db. Table S14. Secondary metabolites genes and clusters identified by SMURF. Table S15. AntiSMASH based functional annotation of secondary metabolite clusters. Table S16. NaPDoS analysis to detect condensation (C) and ketosynthase (KS) domains. TableS 17. Primer sequences for quantitative real-time PCR analyses. Figure S1. k-mer analysis to predict assembly size of P. lilacinum. (ZIP 201 kb) [file 12864_2015_2229_MOESM1_ESM.zip › additional file 1/Additional file 1 fig S1.doc]

**Additional file 1: Figure S1:** k-mer analysis to predict assembly size of *P. lilacinum*
